# Shock Absorption Capacity of High-Performance Polymers for Dental Implant-Supported Restorations: In Vitro Study

**DOI:** 10.3390/dj12040111

**Published:** 2024-04-17

**Authors:** Maria Menini, Francesca Delucchi, Francesco Bagnasco, Domenico Baldi, Luigi Canullo, Paolo Setti, Marco Migliorati, Enrico Simetti, Paolo Pesce

**Affiliations:** 1 Division of Prosthodontics and Implant Prosthodontics, Department of Surgical Sciences (DISC), University of Genova, 16132 Genova, Italy; dafne.1995@libero.it (F.D.); fcbagna5@hotmail.it (F.B.); domenico.baldi@unige.it (D.B.); luigi.canullo@unige.it (L.C.); paolo.pesce@unige.it (P.P.); 2 Private Practice, 16012 Genova, Italy; paolo.setti@unige.it; 3 Orthodontic Division, Department of Surgical Sciences (DISC), University of Genova, 16132 Genova, Italy; marco.migliorati@unige.it; 4 Department of Informatics, Bioengineering, Robotics and Systems Engineering (DIBRIS), University of Genova, 16132 Genova, Italy

**Keywords:** dental implants, shock absorption, high-performance polymers, polyetherketoneketone

## Abstract

Background: Restorative materials might significantly affect load transmission in peri-implant bone. The aim of the present study is to evaluate the shock absorption capacity of two different polymeric materials to be used for implant-supported prostheses. Methods: A masticatory robot was used to compare the shock absorption capacity of veneered and non-veneered polyetherketoneketone (PEKK), Pekkton^®^ivory (Cendres+Mètaux), and the glass fiber-reinforced composite (GFRC), TRINIA^TM^ (Bicon). Five identical sample crowns for each of the three groups were tested. Forces transmitted at the simulated peri-implant bone were recorded and statistically analyzed. Results: The statistical analysis of forces transmitted at the simulated dental implant revealed significant differences between the materials tested and between these materials and zirconia, glass ceramic, composite resin, and acrylic resin. Only differences between PEKK and veneered PEKK and between PEKK and one of the previously tested composite resins were not statistically significant. PEKK samples demonstrated significantly greater shock absorption capacity compared to GFRC. Conclusions: PEKK revealed optimal shock absorption capacity. Further studies are needed to evaluate its efficacy in the case of long-span prostheses with reduced prosthetic volume.

## 1. Introduction

The design and material of implant-supported prostheses greatly affect the long-term success of rehabilitation, contributing to the recovery of masticatory function as well as patients’ quality of life. Usually, frameworks for implant-supported prostheses are made by casting metal alloys or milling either titanium or zirconia [1].

Metal alloys’ high elastic modulus and consequent high stiffness should allow an even distribution of masticatory loads at the supporting implants in the case of multi-unit rehabilitations. However, metal entails high costs and process time. In addition, its adhesive affinity with veneering materials is not optimal and chipping of the aesthetic veneer is not uncommon, with consequential patient discomfort [1]. While lost-wax casting of gold alloys was traditionally considered the gold standard, nowadays, computer-aided design–computer-aided manufacturing (CAD-CAM) is more commonly applied because of its several advantages: reduced manufacturing time, use of industrially prepared materials (virtually defect-free), greater repeatability, and the possibility of a completely digital workflow. The development of CAD-CAM technology has gone hand in hand with the development of materials suited for this application, and among them, the most used are titanium and zirconia.

Titanium, along with its alloys, is also rigid and biocompatible, but it has a high melting point and reactivity, making special equipment necessary for its processing [2].

In order to meet increasing aesthetic demands, metal-free dental materials for CAD-CAM have been developed that include zirconia, ceramics, polymers comprehending high-performance polymers, and glass fiber-reinforced polymers such as the ones tested in the present study.

The use of zirconia prostheses in modern implantology is increasingly common as an alternative to conventional metal-based restorations. Aesthetic concerns lead to the indication of zirconia, due to its favorable biocompatible, biological, and mechanical properties and the possibility of monolithic restorations [3]. 

The principal technical complication of zirconia implant-supported fixed dental prostheses is chipping or fracture of the layering porcelain [4]. A clinical study found a 31.25% porcelain chipping/fracture rate after 2–4 years of function [5]. In addition, some authors suggested caution in employing zirconia frameworks, especially in the case of potential risk factors for mechanical complications (e.g., parafunctional habits), because of zirconia’s high rigidity and strain concentration [6].

According to two recent systematic reviews, zirconia’s high weight and difficult adjustment and polishing, as well as complications such as ceramic veneer chipping and the less frequent framework failure, still represent frequent and unsolved problems [1,7]. 

As an alternative, polymeric materials might be also used for implant-supported frameworks. 

Since the 1980s, there has been an interest in fiber-reinforced composites (FRCs), a group of nonmetallic biomaterials consisting of a resin matrix incorporating fibers made of carbon (carbon fiber-reinforced composites, CFRCs) or glass (glass fiber-reinforced composites, GFRCs). Previously published studies demonstrated the feasibility of using CFRCs or GFRCs for the fabrication of full-arch implant-supported prostheses [1,8]. 

CFRCs’ special features include biocompatibility, aesthetics, low weight, and good static and dynamic strength, especially breaking strength in relation to weight. They are also less expensive than metals. In addition to cohesion agents and coatings (which improve the wettability of the fibers and their adhesion to the matrix), they possess fillers that provide dimensional stability [9,10].

According to a study by Menini et al., prostheses with a carbon fiber-reinforced framework showed an amount of stress transfer to the supporting implants that was intermediate between metal frameworks and full-acrylic prostheses [10].

CFRCs demonstrate optimal biocompatibility and mechanical characteristics, and they appear suitable for the fabrication of frameworks for implant-supported full-arch restorations [11]. 

GFRCs have been also proposed as a framework material for implant-supported prostheses, providing aesthetic advantages compared to CFRCs. GFRCs are employed to fabricate copings, endodontic posts, and frameworks for anterior or posterior crowns and bridges (either teeth or implant-supported), and for cemented or uncemented restorations, such as telescopic crowns. 

FRC restorations can be performed with both analogic or CAD-CAM workflows. When dealing with FRCs, it must be considered that the type of fibers, their disposition, and their manufacturing techniques dramatically affect the mechanical characteristics of the prosthodontic device [12].

In addition, high-performance polymers (HPPs) are available. The HPPs that have entered dentistry are the polyaryletherketones (PAEKs), a family of synthetic semicrystalline thermoplastic polymers including different materials with varying chemical structures [13]. PAEKs are commonly described in terms of an “E” and a “K”, which denote the sequence of ether and ketone group units in the polymer structure. 

The most common PAEKs are polyetheretherketone (PEEK) and polyetherketoneketone (PEKK), although other polymers such as polyetherketone (PEK), polyetherketoneetherketoneketone (PEKEKK), etc., also exist. In their unaltered, unfilled state, these materials are ivory-greyish in color and they can be filled with pigments or reinforcing agents in order to improve their aesthetic and mechanical performances. 

Specifically, Biocompatible High-Performance Polymer (BioHPP) stands out as a subgroup of PEEK, enriched with 20% extra ceramic fillers. This material finds applications in various dental procedures, including the construction of removable partial denture frameworks, removable dentures, obturators, crowns, fixed partial dentures, post-and-cores, and implant abutments [14,15,16]. 

On the other hand, polyetherketoneketone (PEKK) is a synthetic methacrylate-free HPP with a semicrystalline structure. It was first introduced by Bonner in 1962, and since then, it has been used for different industrial and military purposes [17]. Thanks to its several qualities, it was introduced in the medical and dental fields with a wide range of applications [18]. It has excellent mechanical properties, including good compressive and fracture strength, low density, low weight, favorable melting temperature, semiradiolucency, antibacterial activity, an ivory color similar to that of teeth, chemical and wear resistance, and excellent biocompatibility [17,18,19,20,21,22,23,24].

Regarding mechanical properties, PEKK demonstrates a good strength-to-weight ratio and resembles the characteristics of human mineralized tissues. In fact, its density, compressive strength, and modulus of elasticity (Table 1) are comparable to those of dentin and bone. PEKK has an 80% greater compressive strength and better long-term fatigue properties than unreinforced PEEK because of the extra ketone group [22].

Due to the above-mentioned characteristics, PEKK has been taken into consideration as a restorative material for fixed prostheses in order to fabricate both monolithic and bi-layered structures, the latter veneered with composite resin, crowns, and bridges on natural teeth and on implants, inlay cores with or without onlays, and even monolithic posterior crowns [17]. According to a study by Alsadon et al., the fatigue limit of PEKK composite-coated molar crowns was comparable to that of cobalt–chrome and polymethylmethacrylate (PMMA) (750 N) [25].

Several other applications have been documented, including endodontic posts, splinting devices, implant abutments with titanium bases, frameworks for overdentures and removable prostheses, etc. [26,27,28]. PEKK has been also used to fabricate dental implants [17]. 

PEKK can be manufactured in two different ways: injection molding (pressing) or the CAD-CAM milling process. It must be underlined that the surface preparation of PEKK before bonding (a specific procedure combining mechanical retention and chemical bonding) is mandatory in order to obtain acceptable bonding values. According to an in vitro study by Fuhrmann et al., evaluating resin bonding to PAEKs, crystalline and amorphous PEKK exhibited lower values of tensile bond strength than fiber-reinforced PEEK, and the highest values were achieved after conditioning PEKK and PEEK with silica coating and priming [29]. 

Ease of production and repair, resistance to corrosion, possible shock absorption capacity, good aesthetics, low costs, etc., also make polymeric materials like PEKK particularly suitable for implant prosthodontics when compared to other aesthetic materials such as ceramic and zirconia [30]. 

In addition, restorations made of radiolucent polymers such as PEEK or PEKK can significantly reduce CT artifacts and increase image quality compared to titanium and zirconia [31].

However, these new promising materials need in vitro and in vivo investigations to validate their effectiveness.

In particular, the present in vitro study focused on the shock absorption capacity of restorative materials. “Shock absorption” can be considered the capacity to dampen loads, reducing the forces transmitted to underlying structures. Two relevant mechanical properties related to shock absorption are flexural strength and elastic modulus. In fact, an elastic material deforms under a load and distributes the load in an extended time period compared to a more rigid material, reducing the maximum amount of force transmitted [32].

This aspect has to be considered when investigating the eventual application of PEKK as a framework material in implant prosthodontics, especially for full-arch implant-supported rehabilitations and in the case of immediate loading protocols where load control is mandatory to ensure adequate osseointegration avoiding or minimizing the risk of implant micromovements. Proper load control is tied to patient-related factors and to prosthesis design and material [33]. The incorporation of a rigid substructure, effectively splinting the implants together, aims to achieve a uniform distribution of occlusal stress across the abutments and implants. This strategy is intended to mitigate excessive compressive forces and strains on the peri-implant bone, especially crucial during the initial phase following implant insertion, particularly in instances of immediate loading [1,10]. At the same time, the shock absorption capacity of restorative materials might help dampen occlusal loads [34].

Shock absorption is supposed to be different for this innovative polymeric material, compared to alternative polymeric materials (fiber-reinforced composites) and traditional materials (acrylic resin, traditional resin composites, glass ceramic, gold alloys, zirconia).

Therefore, the aim of the present in vitro study was to compare the shock absorption capacity of a high-performance polymer with the shock absorption capacity of a glass fiber-reinforced composite through the use of a masticatory robot. 

The null hypothesis tested was that no differences existed in the shock absorption capacity of the materials tested.

## 2. Materials and Methods

**Figure 1:**
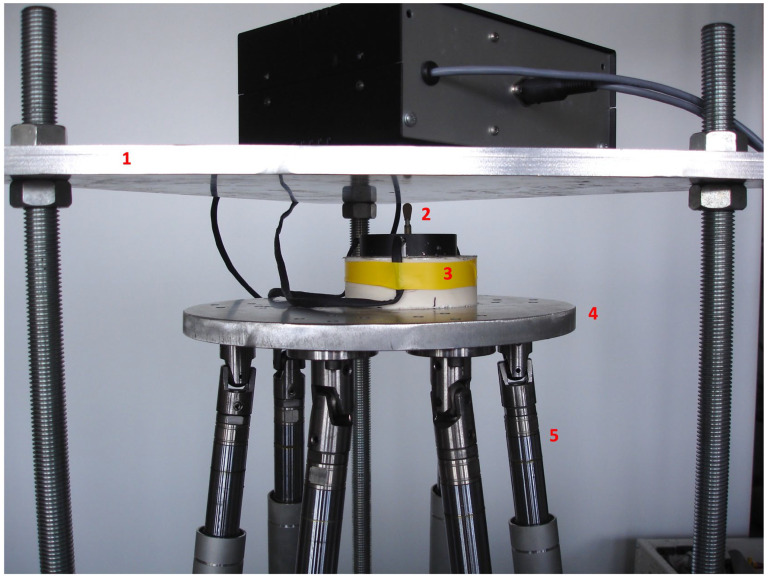
The sensor-equipped masticatory robot employed in the present research. 1. Fixed upper part; 2. pin supporting the sample crowns; 3. sensor-equipped base; 4. mobile platform (Stewart platform) simulating the chewing cycle and masticatory forces; 5. six identical kinetic legs.

The masticatory robot set-up of the University of Genova (Italy) used in previously published papers [34,35] (Figure 1) was used to compare the shock absorption capacity of 2 polymeric materials: the polyetherketoneketone Pekkton^®^ivory (Cendres+Mètaux SA, Biel/Bienne, Switzerland) and the glass fiber-reinforced nanohybrid composite resin TRINIA^TM^ (Bicon, Boston, MA, USA), which consists of 60% multi-directional interlacing glass fibers (woven configuration) and 40% epoxy resin in several layers [16,36,37,38] (Table 1). 

**Figure 2:**
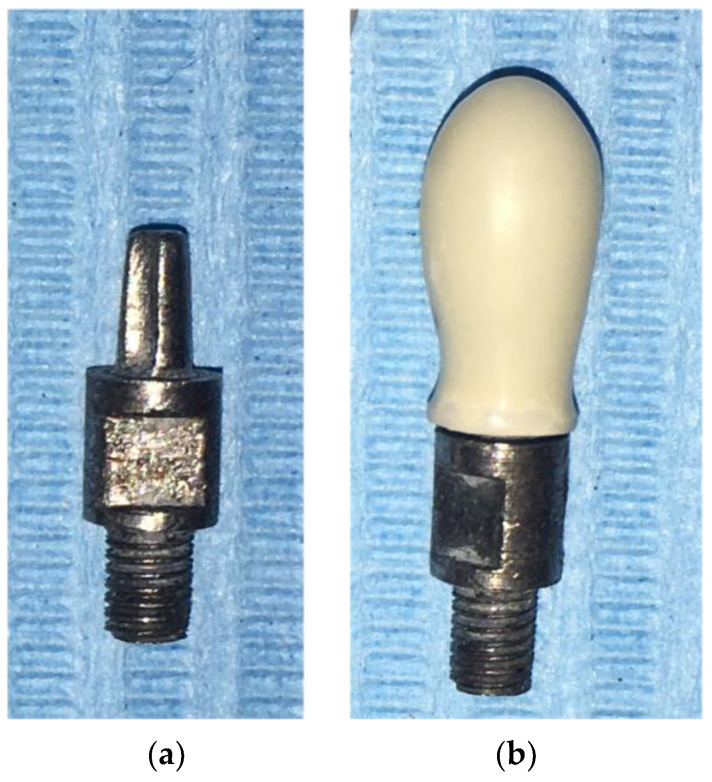
(**a**) Pin simulating the implant–abutment system provided with a groove that matches with the sample crown; (**b**) PEKK crown inserted onto the pin.

The masticatory robot is able to three-dimensionally simulate the masticatory cycle and reproduce the forces exerted during mastication. The movable part of the robot is constituted of a Stewart platform, and it is equipped with a sensorized base that records the loads transmitted at the pin, simulating the implant–abutment system (Figure 2). Sample crowns to be tested are placed on the pin [35] without the use of any luting material.

The start (=end position) position of the robot chewing cycle has the sample crown in contact with the rigid upper part of the robot. The forces transmitted at the base of the pin are recorded during robot occlusion on three axes (*x*, *y*, *z*). 

### 2.1. Sample Crowns

Five identical sample crowns were made of each of the materials to be tested: 5 Pekkton^®^ivory full-contour samples (PI), 5 Pekkton^®^ivory veneered samples (PIV), and 5 TRINIA^TM^ full-contour samples (TR) (n = 15). The sample crowns had a simplified shape, with a semispherical occlusal surface (Figure 2); therefore, they had a single contact point during robot occlusion. At this point, the crowns were 5 mm thick.

Both PI and TR sample crowns were milled. Pekkton^®^ivory was previously available both for pressing and milling. The pressing technology was taken off the market, but CAD-CAM milling blanks are still available and have been used in the present study. PIV crowns were manufactured using milled Pekkton^®^ivory as a core material with thicknesses in the range of IFU recommendations (i.e., 1 mm). The core was veneered using the composite resin anaxBLEND Flow opaquer and dentin (anaxdent GmbH, Stuttgart, Germany). Veneering with composites was standardized using a clear silicone template.

All the crowns had identical shapes and dimensions to the ones tested in previously published studies in order to be fitted on the existing metal pin simulating the implant–abutment system to be screwed into the masticatory robot [34].

### 2.2. Test Set-Up

The samples were numbered for blinding and tested following a random order using the robotic chewing simulator of Genoa University under the same conditions described in previously published papers [34]. All the crowns were positioned in occlusion with the flat fixed upper part of the robot (start position = end position) and were subjected to 100 consecutive chewing cycles. The chewing robot was set to follow the same 3-dimensional trajectory used in previously published studies [34]. The robot always followed this same trajectory independently from the forces exerted. The only variable in the system was the material from which the sample crowns were fabricated.

Thanks to the strain gauges of the sensorized base, the forces transmitted at the base of the pin were recorded. 

### 2.3. Statistical Analysis

Given the high mean difference between the different materials, a total of 3 samples for each material was considered a sufficient sample size, in analogy to previously published studies.

Only vertical loads on the *z*-axis were considered and then processed with MATLAB 6.1 software (MathWorks). The maximum values of the forces (N) for each chewing cycle were recorded, and the mean and standard deviation (SD) of the maximum values of the forces were calculated for each sample crown. These values underwent statistical analysis using the software SAS 9.4. One-way analysis of variance (ANOVA) was used to compare maximum forces, and Welch’s *t*-test, considered in case of unequal variances, was computed. The data recorded in the present investigation were also compared with data reported in a previously published study for different restorative materials (acrylic resin, composite resin, gold alloy, glass ceramic, and zirconia) [34]. Alpha was set at 0.05.

## 3. Results

No fractures nor chippings of the samples occurred during the tests.

All the sample crowns showed a shock absorption behavior similar to the composite resin materials tested in previously published studies [34]. The values of stresses transmitted to the simulated peri-implant bone were far lower compared to zirconia, ceramic materials, and metal alloys.

The resulting vertical loads expressed in Newton are presented in Table 2.

The analysis of variance (one-way ANOVA) revealed statistically significant differences with a *p*-value < 0.0001 and, therefore, the null hypothesis was rejected. Welch’s *t*-test was therefore performed and the outcomes are reported in Table 3.

No considerable differences were found between Pekkton^®^ ivory and Pekkton^®^ ivory Veneered, within a significance level of 5% (*p*-value Pekkton^®^ ivory vs. Pekkton^®^ ivory veneered 6.45%).

On the contrary, TRINIA^TM^ showed higher values of force compared to Pekkton^®^ ivory and Pekkton^®^ ivory Veneered.

Both Pekkton Ivory vs. TRINIA^TM^ and Pekkton^®^ Ivory Veneered vs. TRINIA^TM^ presented statistically significant differences in the forces transmitted at the simulated dental implant with *p*-values < 1%.

**Figure 3:**
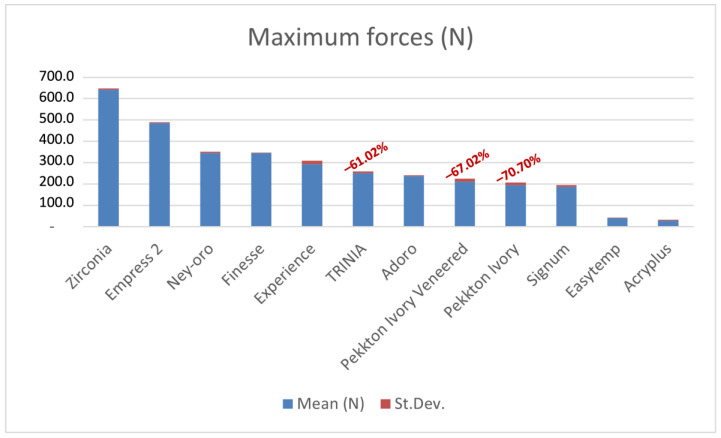
Mean maximum forces (N) of the materials tested in the present study compared with materials tested in a previously published study by the same team of authors [34]. In red: difference in force transmitted compared to zirconia.

Welch’s *t*-test was applied to individually compare Pekkton Ivory, Pekkton Ivory Veneered, and TRINIA^TM^ with each of the materials tested in the previously published paper (Figure 3) [34]. All the comparisons revealed statistically significant differences at 1% except the comparison between Pekkton Ivory and Signum composite resin (*p*-value: 0.2396).

## 4. Discussion

The present investigation sheds light on the shock absorption capacity of two different polymers employed for implant-supported fixed prostheses. Indeed, shock absorption was not directly measured, but the differences in forces transmitted through the crowns at the simulated implant were considered an indication of the shock absorption capacity of the different materials.

Sample crowns were manufactured using PEKK (Pekkton^®^ivory) and a glass fiber-reinforced composite (TRINIA^TM^). The null hypothesis was rejected; in fact, the use of different restorative materials significantly affected the forces transmitted to the simulation of the dental implant. In particular, PEKK, thanks to its structure and low elastic modulus (3–5 GPa), presented a high shock absorption capacity and demonstrated significantly greater shock absorption compared to TR, which might contribute to a reduction in occlusal loads. Only the differences between PI and PIV and between PI and Signum composite material were not statistically significant, probably due to the similar elastic modulus of the two polymers.

Tests were also performed with veneered crowns using Pekkton^®^ivory as the core material and a composite veneering material (anaxBLEND Flow opaquer and dentin) in order to be closer to real situations. In fact, PEKK requires veneering due to its low translucency and grayish color. The bond strength of composite veneering materials is one of the aspects of PEKK that needs further evaluation in future studies [18].

In fixed prosthodontics, the selection of the framework material plays a pivotal role in ensuring long-term clinical success. The framework not only supports the artificial teeth but also effectively transfers loads to the substructures, including implants and the surrounding peri-implant bone [39]. Implant-supported frameworks are most commonly made by casting metal alloys or milling titanium or zirconia, but polymeric materials are also becoming increasingly popular and one of their claimed advantages is their shock absorption capacity, which might reduce the forces transmitted at the implant components and at the peri-implant bone.

Jovanovic et al. found that glass fiber-reinforced resin-based materials exhibit a significant reduction in the impact of functional loads on implants, up to 50% when compared to ceramic-reinforced resin-based materials. This is attributed to their lower elastic modulus (18.8 GPa), which closely resembles that of dentin (18.6 GPa) [16]. 

In the in vitro study by Omaish et al., the microstrain values recorded around implants restored with TRINIA^TM^ were also significantly lower than those restored with bioactive high-performance polymer (BioHPP) when a static load was applied, with considerably higher microstrain when the implant abutment angulation increased [37]. Reduced stress transmission might reduce the risk of biological and technical complications in implant-supported rehabilitations.

Regarding PEKK, in vitro studies and short-term clinical reports have evaluated its use in dentistry for implant-supported dental prostheses with favorable outcomes [40,41]. However, little is known yet about the mechanical response of PEKK as a framework material for implant-supported full-arch prostheses. 

A finite element analysis by Shash et al. investigated the effect of polymeric frameworks (e.g., CFR-PEEK 30%, CFR-PEEK 60%, PEKK, and PEEK) as alternatives to titanium frameworks in implant-supported full-arch fixed prostheses. PEKK frameworks slightly reduced the stresses on bone tissues, especially in low-density bone; however, they increased the mucosal stress (even if these values did not exceed the pain threshold value) [42]. CFR-PEEK 60% distributed the loads in a similar manner to the titanium framework. The authors concluded that polymeric frameworks can offer improved performance and aesthetics, greater design freedom, production of lighter prostheses, reduced overall cost, and reduced manufacturing and mechanical problems compared with titanium (Shash et al., 2023).

According to a 3D-FEA by Lee et al., simulating a full-arch prosthesis supported by four maxillary implants, PEKK structures showed lower implant and tissue stress under compressive strain and higher stress under tensile strain. Therefore, the authors suggested that PEKK, being a resilient structure, should be limited in some areas; conversely, a more rigid material such as titanium or zirconia can provide favorable stress distribution and safety of the implant prosthodontic components [43].

A recent study by Villefort et al. employed finite element analysis to compare the behavior of PEKK and PEEK prosthetic frameworks used in the all-on-four concept. It was observed that PEKK, thanks to its superior shock absorbance, induced a lower stress concentration on the prosthetic screw and the prosthetic base. According to the authors, this would clinically represent lower risks of fractures on the acrylic base and screw loosening [20]. 

Thanks to their shock absorption potential, resilient veneering materials, such as acrylic or composite resin, have been proposed for coating rigid metal frameworks in traditional treatment protocols for full-arch implant-supported rehabilitations [44]. 

Results previously reported by Menini et al. [34] have shown that composite and acrylic resins absorb shocks from occlusal forces significantly better than ceramic and zirconia. Similarly, in the present study, both PEKK and GFRC transmitted significantly lower stresses to the simulated implant compared to ceramic and zirconia. This would allow a reduction in loads at the bone–implant interface [34]. 

However, the in vitro study by Ogawa et al. [45] demonstrated that bending moments were reduced in full-arch implant-supported prostheses when a titanium framework was used compared to a GFRC framework or a full-acrylic prosthesis. A rigid framework, presenting reduced bending moments under loads, allows for a more even distribution of occlusal loads over the supporting implants [18]. As a consequence, the presence of a rigid framework, combined with the shock-absorbing effect of the veneering material, can be considered by the authors to be an optimal option for controlling occlusal loads. In this regard, it must be underlined that the rigidity of the framework depends not only on the material of which it is made but also on its design and, in particular, on its length and thickness. Therefore, a material with a greater elastic modulus might be indicated when the prosthetic volume is reduced and the prosthesis presents long spans, while the advantages of more elastic polymeric materials might be favorably exploited in the case of greater prosthetic volumes. In fact, in this case, the light weight of polymeric materials increases patients’ comfort compared to prostheses with a zirconia or metal structure. In addition, the ease of repairing and relining polymeric devices makes them particularly suitable and versatile for prosthodontic applications.

In addition, the possibility of milling FRC as well as PAEK devices allows the application of completely digital workflows and improves the standardization of the mechanical characteristics of the final devices, reducing the risks of possible defects related to traditional manufacturing techniques, which might affect the mechanical behavior of the prosthesis [30].

Interestingly, the studies mentioned above applied different methods to test the stress transmission and shock absorption capacity of restorative materials. For this reason, a comparison of the outcomes of different studies is not possible. In fact, a standard method to test shock absorption is not available, and further studies should focus on the development of a protocol to test the shock absorption of dental materials. 

As explained in detail in our previous study [34], the in vitro model applied in the present investigation has a number of limitations in reproducing the three-dimensional clinical reality of masticatory function. It was not possible to use an implant analog placed in a resilient bone simulation. Only three materials were compared in terms of one property (shock absorption), without aging, thermal cycling, or cyclic loading, which are important in order to simulate long-term clinical performance. 

In fact, to make our data comparable with those obtained with other materials in our previous studies [34], the same methodology as the previously published studies was applied [34]. 

In addition, only single crowns were tested in order to evaluate the shock absorption capacity. In fact, the masticatory robot in its present form does not allow the testing of multi-unit prostheses. Different outcomes should be expected when testing single crowns rather than multi-unit prostheses due to the possible flexure of the bridge span. If the aim was to evaluate the mechanical behavior of the tested materials for the realization of frameworks, a multi-unit rehabilitation with long-span frameworks should have been simulated. In particular, the authors underline that the use of PEKK and glass fiber-reinforced composites as framework materials should be carefully considered based on the prosthetic volume available. In fact, the thinner the framework and the longer the bridge span, the greater its flexure and possible bending moments on implant–prosthodontic components. Therefore, in the case of multi-unit rehabilitations, the outcomes could be different in terms of load transmitted to the dental implant and peri-implant bone.

Another limitation might be the fact that only vertical loads were recorded and analyzed. In fact, in the present study set-up, where sample crowns were brought into occlusion with the flat upper part of the robot (without a simulation of antagonist teeth), vertical loads were predominant and transversal loads were negligible. In addition, a previous paper [35] demonstrated that the percentage difference of force recorded using different materials was superimposable on the three axes (*x*, *y*, and *z*): data for the three axes were redundant. In addition, variations in occlusion, patient characteristics, antagonist arch, etc., should be considered as they might affect the outcomes.

On the other hand, the intrinsic advantages of in vitro studies include the possibility of reducing the variables possibly affecting and confounding the outcomes of a clinical scenario. The chewing simulator herein applied has been validated in a previous study [20] and demonstrated effectiveness and repeatability in tests. Moreover, the masticatory robot has the advantage of overcoming some of the limitations of other in vitro studies that rely, for example, on finite element analysis, undermined by the simplification of results inherent in the virtual simulation itself [20].

In conclusion, according to the results of the present research, where single crowns were tested, PEKK significantly reduced the stresses transferred to the simulated peri-implant bone compared to materials with a greater elastic modulus. This makes it a promising material for metal-free implant-supported prostheses. However, its behavior in the case of multi-unit restorations needs to be evaluated in further studies, also in relation to the prosthetic volume and length of the span.

Based on the present outcomes and on data reported in the literature, further investigations and especially long-term clinical studies are needed before recommending its application as a restorative material for implant-supported full-arch prostheses, especially in the case of long-span prostheses with reduced prosthetic volumes where the risk of prosthesis flexure might indicate the use of more rigid framework materials.

## Figures and Tables

**Table 1:** Table summarizing the main mechanical properties of the materials tested.

Material Property	TRINIA^TM^	Pekkton^®^ivory
Flexural strength	393 MPa	200 MPa
Flexural modulus of elasticity	18.8 GPa	5.1 GPa
Tensile strength	169 MPa	119 MPa
Compressive strength	339 MPa	246 MPa
Rockwell hardness (R-scale)	870 HV	33 HV
Density	1.68 g/cm^3^	1.4 g/cm^3^
Fracture strength	9.7 MPa m^1/2^	115 MPa
Water absorption	0.03%	8.7 μg/mm^3^

**Table 2:** Table reporting mean maximum occlusal forces (N) recorded during 100 masticatory cycles of the masticatory robot using sample crowns made of PEKK and glass fiber-reinforced composites.

Pekkton^®^ivory			Pekkton^®^ivory Veneered			TRINIA^TM^		
Mean ± SD (N)	Min (N)	Max (N)	Mean ± SD (N)	Min (N)	Max (N)	Mean ± SD (N)	Min (N)	Max (N)
194.454 ± 10.544	169.962	217.816	211.640 ± 12.437	189.975	239.053	250.203 ± 7.954	237.622	286.513

**Table 3:** Table reporting the results of Welch’s *t*-test highlighting statistically significant differences in the forces transmitted using different materials.

Material 1	Material 2	Welch’s *t*-Test *p*-Value
Pekkton Ivory Veneered	Zirconia	<0.0001
Pekkton Ivory Veneered	Empress 2	<0.0001
Pekkton Ivory Veneered	Ney-oro	<0.0001
Pekkton Ivory Veneered	Finesse	<0.0001
Pekkton Ivory Veneered	Experience	<0.0001
Pekkton Ivory Veneered	TRINIA	<0.0001
Pekkton Ivory Veneered	Adoro	0.0165
Pekkton Ivory Veneered	Pekkton Ivory Veneered	<0.0001
Pekkton Ivory Veneered	Pekkton Ivory	<0.0001
Pekkton Ivory Veneered	Signum	0.0167
Pekkton Ivory Veneered	Easytemp	<0.0001
Pekkton Ivory Veneered	Acryplus	<0.0001
Pekkton Ivory	Zirconia	<0.0001
Pekkton Ivory	Empress 2	<0.0001
Pekkton Ivory	Ney-oro	<0.0001
Pekkton Ivory	Finesse	<0.0001
Pekkton Ivory	Experience	<0.0001
Pekkton Ivory	TRINIA	<0.0001
Pekkton Ivory	Adoro	0.0012
Pekkton Ivory	Pekkton Ivory Veneered	<0.0001
Pekkton Ivory	Pekkton Ivory	<0.0001
Pekkton Ivory	Signum	0.2396
Pekkton Ivory	Easytemp	<0.0001
Pekkton Ivory	Acryplus	<0.0001
TRINIA	Zirconia	<0.0001
TRINIA	Empress 2	<0.0001
TRINIA	Ney-oro	<0.0001
TRINIA	Finesse	<0.0001
TRINIA	Experience	0.0002
TRINIA	TRINIA	<0.0001
TRINIA	Adoro	0.018
TRINIA	Pekkton Ivory Veneered	<0.0001
TRINIA	Pekkton Ivory	<0.0001
TRINIA	Signum	<.0001
TRINIA	Easytemp	<0.0001
TRINIA	Acryplus	<0.0001

## Data Availability

The data that support the findings of this study are available from the corresponding author, M. Menini, upon request.
